# Example study for granular bioreactor stratification: Three-dimensional evaluation of a sulfate-reducing granular bioreactor

**DOI:** 10.1038/srep31718

**Published:** 2016-08-19

**Authors:** Tian-wei Hao, Jing-hai Luo, Kui-zu Su, Li Wei, Hamish R. Mackey, Kun Chi, Guang-Hao Chen

**Affiliations:** 1 Department of Civil & Environmental Engineering, The Hong Kong University of Science and Technology, Clear Water Bay, Kowloon, Hong Kong, China; 2 School of Civil Engineering and Water Conservancy, Hefei University of Technology, Hefei, China; 3 College of Science and Engineering, Hamad bin Khalifa University, Education City, Doha, Qatar; 4 Water Technology Lab, The Hong Kong University of Science and Technology, Clear Water Bay, Kowloon, Hong Kong, China; 5 Hong Kong Branch of Chinese National Engineering Research Center for Control & Treatment of Heavy Metal Pollution, HKUST, Clear Water Bay, China

## Abstract

Recently, sulfate-reducing granular sludge has been developed for application in sulfate-laden water and wastewater treatment. However, little is known about biomass stratification and its effects on the bioprocesses inside the granular bioreactor. A comprehensive investigation followed by a verification trial was therefore conducted in the present work. The investigation focused on the performance of each sludge layer, the internal hydrodynamics and microbial community structures along the height of the reactor. The reactor substratum (the section below baffle 1) was identified as the main acidification zone based on microbial analysis and reactor performance. Two baffle installations increased mixing intensity but at the same time introduced dead zones. Computational fluid dynamics simulation was employed to visualize the internal hydrodynamics. The 16S rRNA gene of the organisms further revealed that more diverse communities of sulfate-reducing bacteria (SRB) and acidogens were detected in the reactor substratum than in the superstratum (the section above baffle 1). The findings of this study shed light on biomass stratification in an SRB granular bioreactor to aid in the design and optimization of such reactors.

Biological treatment is widely implemented for conversion of organic compounds and nutrients because of its environmental and economic merits. The major constraint on biological treatment processes lies in biomass retention and its separation efficiency from reactor effluent. As a solution, biomass granular aggregates have been developed and investigated intensively in the past 30 years^1^,^2^.

Since sludge granulation was first observed in anaerobic methanogenic bioreactors^1^, it has been developed across many bioprocesses including organic and ammonia oxidation^3^,^4^, enhanced biological phosphate and nutrient removal^5^, anammox^6^ and sulfate reduction^7^. Granular sludge bioreactors possess excellent capabilities of liquid-solid separation and biomass retention and are resilient against variations in pollution loads and other environmental conditions^8^,^9^,^10^,^11^.

However, vertical stratification of biomass is often observed in various types of granular sludge reactors including anaerobic, anoxic and aerobic bioreactors^12^,^13^,^14^,^15^. The stratification is viewed as a disadvantage of granular reactors; a decrease in hydrodynamic shear in the upper section of the reactor causes the top layer of sludge to become unstable, thereby affecting the reactor operation^16^,^17^,^18^,^19^. More importantly, the stratification reduces the overall treatment capacity^20^,^21^ due to conglomeration of granules and hydraulic short-circuiting^12^. Many attempts have thus been made to reduce/eliminate stratification in anaerobic or anoxic gas-producing (CH_4_, H_2_ and N_2_) granular sludge reactors^12^. Hydrodynamics plays a key role of reducing stratification by increasing the mixing of granules of different sizes and densities and by reducing the vertical substrate gradient in the reactor, thereby promoting more homogenous granule growth. The effects have been well demonstrated by internal circulation anaerobic (ICA) reactors which can have organic loading rates 3–5 times higher than that of UASB reactors through applying a higher superficial liquid velocity and gas velocity^22^. Stratification issues in non-gas-producing anaerobic systems such as sulfate-reducing granular sludge reactors^23^ could be more serious due to the absence of gas bubble mixing in the reactor. Such granular sludge reactors are used for processing sulfate-laden wastewaters produced by various industries^24^ and from the utilization of seawater in coastal areas for toilet flushing as an alternative water resource^25^.

No methane production has been confirmed in a biological sulfate reduction (BSR) granular sludge reactor treating saline municipal sewage generated from seawater toilet flushing at a chemical oxidation demand (COD)-to-sulfate ratio of approximately 0.5^23^. This feature makes SRB granules much denser than methanogenic or hydrogen-producing granules^26^, and hence, stratification of sludge tends to more significant than in other anaerobic granular sludge reactors. Minimizing stratification is key to optimizing the reactor design and/or operation. Therefore, this study systematically investigates a granular sludge sulfate-reducing upflow sludge bed (SRUSB) with the aims of determining the vertical functional zones; identifying the internal hydrodynamic flow pattern through a 3-D computational fluid dynamics (CFD) model^27^; and exploring the vertical variations in microbial community structure along the height of the reactor. Such information will enhance our understanding of the stratification of non-gas-producing granules to facilitate the design and operation of granular SRUSB bioreactors.

## Results and Discussion

The COD removal efficiency of the SRUSB was initially above 84% but gradually dropped to approximately 70% by day 160 (Fig. S1a in the Supplementary Information (SI)) at which time the reactor short-circuited (Fig. S1b). To alleviate this issue two baffles were installed in the SRUSB. The motivation was to increase mixing and create multiple compartments in the reactor to simulate continuously stirred tank reactors.

### Organic degradation profile

**Figure 1:**
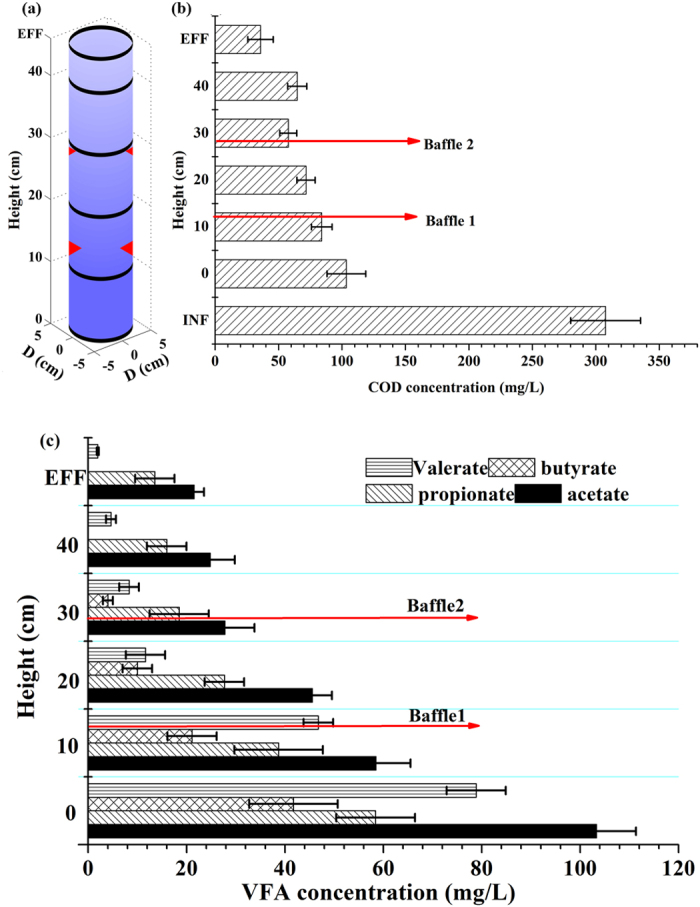
Vertical profile of organic degradation along the height of the SRUSB reactor: (**a**) layer locations for sampling, (**b**) COD and (**c**) VFAs.

To examine the organic degradation profile along the height of the reactor, 9–15 samples were taken at three time points. Detailed data of each layer are shown in Fig. S2. Average soluble COD and volatile fatty acid (VFA) profiles were obtained and are shown in Fig. 1. As expected, COD decreased progressively as the influent passed through the granular sludge bed. COD in the influent was around 320 mg/L on average, and was subsequently diluted by 5Q (Q = influent flow) recirculation (Fig. 1b). The trend of gradually declining COD along the reactor height was observed except between the heights of 30 and 40 cm. In this zone the measured COD actually increased although there was no statistical difference between the two levels using the Student t test (α = 0.05). An average of 36 mg/L soluble COD and 71 mg/L total COD were left in the SRUSB effluent.

The highest VFA level appeared at the bottom of the SRUSB reactor, with approximately 100 mg/L acetate (~40 mg/L came from the influent) representing the main component of VFAs produced from the acidogensis/fermentation of the bottom organics. Butyrate was completely consumed within the reactor and valerate comprised only a minor fraction of effluent VFA. Acetate and propionate became the main organics in the effluent, each contributed around 50% of soluble effluent COD (Fig. 1c) with approximately 40–50% of the remaining effluent COD in particulate form. The metabolic pathway of VFA degradation by SRB can be categorized into two divisions: those that oxidize organics incompletely to acetate and those that oxidize organics to CO_2_ directly. The VFAs such as valerate, butyrate and propionate can be consumed by incomplete and complete organic oxidizers through various pathways (e.g., typical β-oxidation, modified citric acid cycle, etc.)^28^ and the generated acetate also can be degraded by both SRB types via a modified citric acid cycle and the Acetyl-CoA pathway^29^. The formation of acetate is an intrinsic feature under sulfate- or sulfite-reducing conditions due to the co-existence of complete and incomplete VFA oxidizing SRB. For the known 40 SRB genera, 55% cannot metabolize the acetate, moreover, sulfidogenic bioreactors are commonly dominated by *Desulfovibrio, Desulfobulbus*, *Desulfomicrobium* and *Desulfobacter*. Among them, *Desulfovibrio, Desulfobulbus* and *Desulfomicrobium* are incomplete organic oxidizers^29^. This causes the observed rate of acetate uptake by SRB to be lower than the acetate production rate and thus acetate degradation becomes the rate-limiting step in sulfidogenic processes^30^. The unexpectedly high propionate in the effluent could also be the result of incomplete VFA degradation as some SRB species belonging to the genus *Desulfovibrio* have been shown to degrade particular organics to a final product consisting of equal parts acetate and propionate while also being unable to use either acetate or butyrate as substrate^29^.

In general, the degradation patterns of VFAs and COD are in quantitative agreement. However, unlike COD, the average concentration of VFAs in the layer at 40 cm (acetate = 25 mg/L; propionate = 16 mg/L) was lower than that in the layer at 30 cm height (acetate = 27 mg/L; propionate = 19 mg/L; butyrate = 4 mg/L). This confirms that VFAs did not to contribute to the COD concentration in the layer at 40 cm. Hence, the observation of unchanging COD is a localized phenomenon, and which could be caused by macro-molecular organics (e.g., residual endogenous cells or microbial byproducts such as proteins or lipids) generated from the baffle to adjacent zones via hydrolysis/fermentation due to high sludge accumulation. Such behavior could be mitigated by adjusting the hydrodynamics or baffle locations/size.

### Sulfur vertical profile

**Figure 2:**
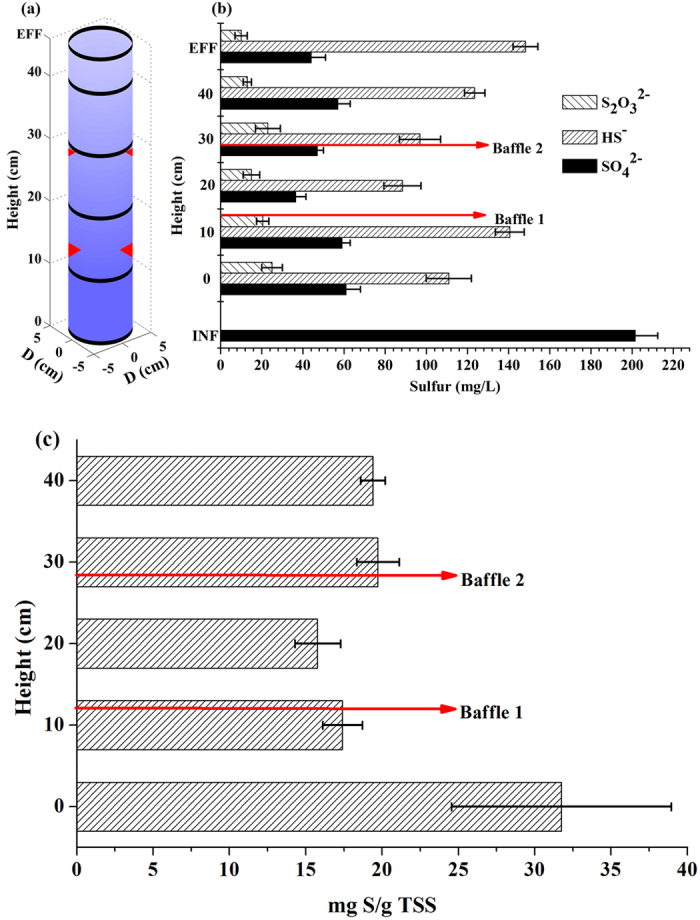
(**a**) Layer locations for sampling, (**b**) sulfate, thiosulfate and total dissolved sulfide generation profiles, and (**c**) poly-S/S^0^ accumulation profile along the height of the SRUSB reactor.

As a result of the internal recirculation the liquid at the bottom of the SRUSB reactor contained sulfate (average 61 mg S/L), dissolved sulfide and thiosulfate. As shown in Fig. 2b the thiosulfate concentration was relatively stable along the height of the reactor (approximately 20 mg S/L) although it dropped slightly in the effluent. The average dissolved sulfide concentration increased from 111 mg S/L at the bottom of the SRUSB reactor to 141 mg S/L at 10 cm height. However, neither sulfate nor thiosulfate reduction was observed within this zone of 0–10 cm (Fig. 2b). Beyond this zone sulfate was decreased from 61 to approximately 40 mg S/L through the sludge layers. However, increased dissolved sulfide concentration, pH and alkalinity (Fig. S3), as well as the depletion of VFAs between the bottom of the reactor and 10 cm height all indicate that sulfate reduction occurred within this zone (0–10 cm). Electron acceptors were hypothesized to come from other sulfur sources, such as accumulated elemental sulfur and/or poly-S/S^0^, which could be produced by sulfide oxidation bacteria (SOB e.g., *Prosthecochloris*)^31^ and can be easily utilized by SRB. These acceptors can serve as both electron donors and acceptors to produce sulfide and sulfate^32^. Measurements of the accumulated poly-S/S^0^ along the height of the reactor (Fig. 2c) indicated nearly 32 mg S/g total suspended solids (TSS) of sulfur accumulated in the sludge at the bottom of the reactor, while in other layers, it was approximately 17 mg S/g TSS on average. Consequently, further batch tests were conducted with granular sludge taken from the reactor bottom and placed in sulfate-free synthetic sewage. After 5 h anaerobic reaction 15–23 mg/L sulfide was detected confirming the use of elemental sulfur and/or poly-S/S^0^ in the bottom zone rich in these compounds.

### pH, alkalinity and hydrophobicity

Heterotrophic sulfate reduction can be expressed by Equation 1^33^.


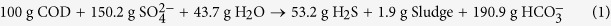


Two moles of bicarbonate are generated with one mole of sulfate reduced according to this equation. Thus the pH and alkalinity could be used as an indicator of the bioreactor performance to some extent. The pH at the bottom of the bioreactor was about 5.6 due to the acidogenic activity (Fig. S3a), which provides sufficient intermediates such as acetate, propionate, butyrate, and lactate for sulfate reduction. With sulfide and bicarbonate generation, the pH and alkalinity increased gradually along the height of the reactor and finally reached plateaus (pH = 7.8, alkalinity = 577 mg CaCO_3_/L) at mid-height of the reactor (Fig. S3b).

The hydrophobicity of the bottom granular sludge was approximately 45% which is close to that of the inoculum and matches the reaction of pH, since most acidogens are hydrophilic^34^. It then increased with reactor height to reach a maximum of 68% in the superstratum sludge (Fig. S4). In the substratum, the lower hydrophobicity of the granular sludge favored the contact/cohesion between substrate and organisms. The 23% hydrophobicity increase of the superstratum granules on the other hand was conducive to biomass separation from the effluent. The hydrophobicity variation of the granular sludge was not only dictated by the bacterial community and surface extracellular polymeric substances (EPS) content, but also influenced by the aqueous chemical composition and hydraulic conditions^35^, which are further investigated below.

### Internal hydrodynamics visualization

**Figure 3:**
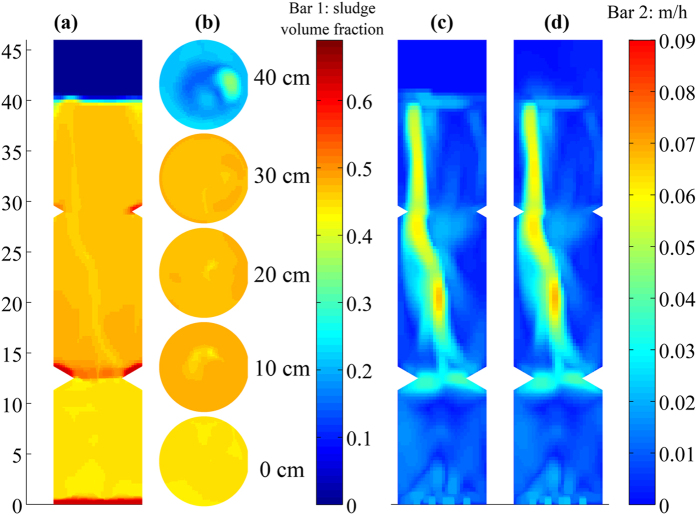
Transient model predictions of the laboratory-scale SRUSB during steady-state operation: (**a**) sludge volume fraction contour, (**b**) sludge volume fraction contours at each reactor cross-section, and (**c**) sludge and (**d**) water velocity magnitude contours.

With the installation of baffles, the performance of the reactor was improved (Fig. S1), and the internal flow pattern/hydrodynamics was revealed. A 3-D numerical simulation was conducted for the steady state granular sludge SRUSB with a structured numerical grid of 602,464 cells. Figure 3a shows the solid phase holdup distribution in the SRUSB. The highest sludge concentration appeared in the layer at 5–15 cm adjacent to baffle 1. The presence of this baffle created a turbulent flow improving sludge mixing and alleviating the short-circuiting. The sludge volume fraction gradually declined along the height of the reactor (Fig. 3a,b), showing a relatively even expansion of the sludge bed.

The simulated flow pattern within the bioreactor is indicated by the contours of velocity magnitude (Fig. 3c,d). The velocity of both sludge and water dramatically increased once the two had passed through baffle 1, after which the flow pattern developed rapidly. Granule and water velocity contours revealed continuous and even hydraulic mixing throughout the sludge zone in contrast to other reports where mixing intensity declined with height^35^ demonstrating the effectiveness of the baffles. For granular bioreactors certain back-mixing is preferred^36^, as it not only relieves the stratification, but also changes the microbial kinetics from zero to a first order at low substrate concentrations. Hence controllable mixing is preferred to enhance the contact between biomass and substrates^36^. On the other hand, contribution of excessive internal mixing provides limited or even detrimental effects on treatment performance. Mixing only needs to meet a level sufficient to ensure mass transfer of substrate through the granule while increased mixing increases energy consumption and increases biomass loss from the reactor.

The vertical profile of simulated granules concentration was obtained from the profile of the sludge volume fraction (Fig. S5). The predicted profile showed a similar trend to measured TSS concentrations at different heights (Fig. S5). The sludge concentration at 10 cm was lower than that in the vicinity of baffle 1 (13 cm) where the sludge concentration reached 24.3 g/L (measured). The regular triangle baffle 1 which protruded by 2 cm reduced the reactor diameter by 45% hindering sludge settlement and boosting the upflow velocity. This resulted in a small compartment with a relatively low sludge concentration below baffle 1, as verified by the simulation result shown in Fig. 3a.

The hydrodynamics simulation presented an actual hydraulic retention time (HRT) of 9.8 min for the SRUSB, which matches that calculated with the 5-Q recirculation (10 min). Therefore, the COD removal rate along the height of the reactor could be determined from the COD degradation profile and the upflow rate. The COD removal rates were 7.3, 10.7 and 4.5 mg/L/min in the bottom (below baffle 1, 0–13 cm), middle (between the two baffles, 13–30 cm) and top layers (above baffle 2), respectively. With such a reactor configuration, the middle layer was the main zone responsible for COD removal via sulfate reduction. This behavior is in accord with conventional UASB reactors, while the bottom layer of the SRUSB also played an important role in fermentation and COD removal in addition to clarification in comparison with the UASB^35^.

### Microbial quantification and analysis of the granular sludge

Although completely-mixed microbial samples have been examined^26^,^31^, to further understand functional zones within the bioreactor, 16S rRNA gene of the SRB granules in the substratum and superstratum were analyzed by pyrosequencing (SRR3233650 and SRR3233651). Rarefaction analysis, based on OTUs at 3%, 5% and 10% dissimilarity, is depicted in Fig. S6.

**Figure 4:**
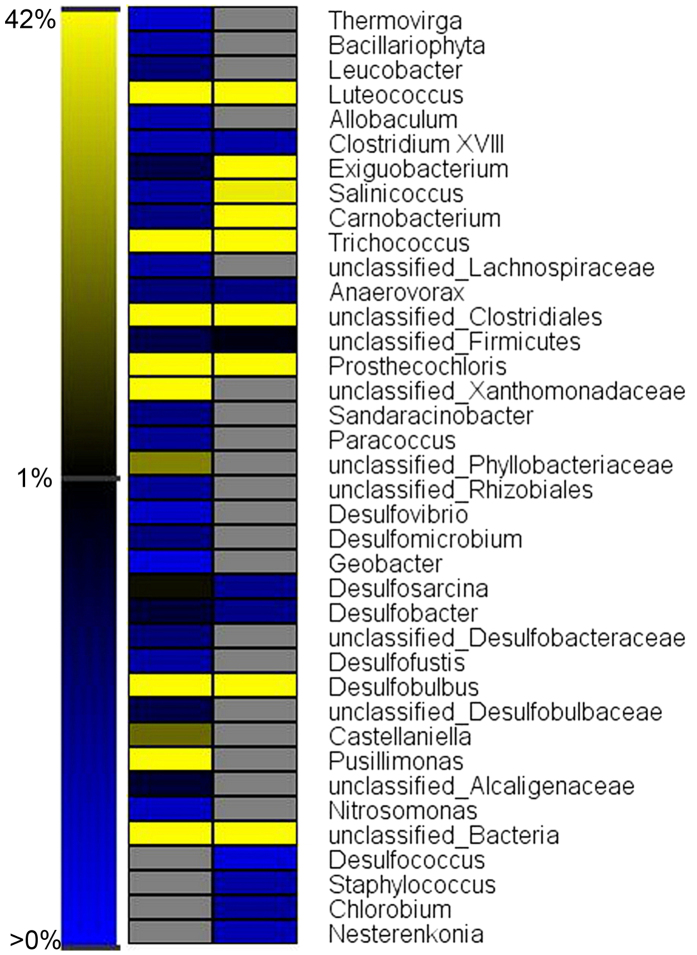
Taxonomic classification of bacterial 16S rRNA reads retrieved from substratum and superstratum samples at the genus level using the RDP classifier with a confidence threshold of 80%.

The microbial analysis at the genus level and sequence abundance greater than 0.2% is illustrated in Fig. 4. More than 15 genera observed in the superstratum granules (above baffle 1) were not present in significant quantities in the substratum granules (below baffle 1). Moreover, the SRB genus abundance in the substratum (41.4% of the total sequences) was less than that of the superstratum (44.5%) as shown in Table 1. The slightly higher abundance of SRB above baffle 1 is consistent with the increased sulfate reduction rated occurring in the reactor superstratum, as determined by the simulated results and the degradation profile of organics. However, a higher SRB diversity was observed within the substratum biomass (Table 1), which could be ascribed to the presence of abundant organics including those in more complex forms prior to their reduction to simple VFAs. For instance, SRB genera *Desulfovibrio, Desulfomicrobium* and *Desulfofustis* were only detected in the substratum. They perform incomplete oxidation of organic compounds (e.g., sugar, long-chain fatty acids, lactate, butyrate and propionate) to acetate^29^. Genera *Desulfosarcina* and *Desulfobacter* were detected from both superstratum and substratum, which metabolize the organics with the end product of CO_2_. A further factor influencing the difference in SRB diversity between the substratum and superstratum is the variation in pH from 5.6–6.7 at the base of the reactor to 7–7.8 in the upper regions (Fig. S3a). Generally, the optimum pH for growth of most SRB is 5–9^37^, and highest rate of sulfate reduction can be achieved at pH of 6.5–7.4^38^, which could contribute to current results. Therefore, optimization of synergism condition between sulfate reduction and acidification should be considered in the reactor operation.

From the vertical profiles of organics consumption and pH variation (Fig. 1 and Fig. S3a), vigorous acidogensis and fermentation are deduced to have occurred at the reactor bottom. This is also supported by the microbial analysis. A higher abundance of acidogen-related genera including *Luteococcus* (1.4%), *Trichococcus* (10.4%) and *Comamonas* (5.8%) was detected in the substratum zone than in the superstratum zone (*Luteococcus* 1.4%, *Trichococcus* 12% and *Comamonas* 0%). *Luteococcus* is mesophilic lactic streptococci that can produce lactic acid via fermentation^39^. Lactate has been shown to be a favorable substrate for SRB metabolism in terms of energy and biomass production^40^.

The genus *Prosthecochloris*, detected at high proportions in the superstratum (19%) and substratum (8.4%), are phototrophic obligate-anaerobes utilizing sulfide as a photosynthetic electron donor and generating sulfur globules that are deposited outside the cells^41^. As depicted in Fig. 2c, the generated sulfur accumulated in the system. Sulfur-utilizing SRB genera, correspondingly *Desulfofustis* (0.39%) and *Desulfobacter* (7.8%), were determined in the reactor substratum. These findings can assist in the interpretation of sulfur reduction in the substratum.

### Baffle relocation in the SRUSB

The unexpected stratification and hydraulic short-circuiting of the SRUSB system was mitigated by installing two baffles, although this did cause some sludge accumulation in the vicinity of the baffles. Based on the microbial community, pH/alkalinity changes, organic components and consumption rates (Table 2), the zone between the two baffles is considered the major zone responsible for organics removal, and the substratum layer of the bioreactor is considered as an acidification zone where readily biodegradable substrates are generated. Therefore, the baffle location was optimized experimentally, by replacing the two baffles by a single baffle (baffle 2) at the height of 20 cm to separate the two bioprocess zones. The optimized baffle location could: 1) enlarge the acidification zone; 2) reduce the sludge accumulation area; and 3) maintain a relatively gentle mixing inside the reactor (reduced back-flow mixing). The bioreactor with the relocated baffle under a sludge concentration of approximately 19 g/L (sludge was withdrawn for other testing purposes during this study) showed a slight improvement in organics removal corresponding to an average soluble COD concentration of 26–30 mg/L in the effluent (Fig. S7). This represents an improvement of approximately 20% compared to the COD concentration of 36 mg/L in the two-baffle system. The one-baffle system is also easier to construct.

## Conclusions

Stratification and hydraulic short-circuiting of a SRUSB were studied and mitigated by installing two baffles in the SRUSB. The 3-D investigation of the internal flow pattern and performance measurement of each sludge layer confirmed improved treatment (15% improvement in terms in organics removal) after the installation of baffles. Sludge distribution was generally homogeneous except for some accumulation of biomass in the vicinity of the baffles.

Microbial analysis and chemical analysis in the baffled SRUSB revealed an acidification zone in the reactor substratum below baffle 1 which was dominated by acidification-related genera (*Trichococcus*, *Luteococcus* and *Comamonas*) comprising 17.6% of retrieved sequences and a more diverse SRB community than that in the superstratum. COD removal was more prominent in the superstratum above baffle 1. The generated poly-S/S^0^ accumulated at the reactor bottom where it was used as an electron acceptor for sulfate reduction in the lower section of the reactor (0–13 cm). An alternative baffle layout based on the hydrodynamic simulation and microbial community analysis was further tested with a single baffle to generate the two observed process zones. This provided a further, minor improvement in overall performance.

## Materials and Methods

### Reactor operation, synthetic wastewater and granular sludge

A 3-liter lab-scale granular sludge SRUSB reactor was operated using synthetic saline sewage for more than 600 days. The synthetic saline sewage is prepared according to Hao *et al*.^7^, in which total COD is equivalent to the biochemical oxygen demand (BOD) and consists of glucose (40%), sodium acetate (40%) and yeast extract (20%). The sludge concentration reached 22 g/L under an HRT of 1 hr after 120 days of operation. Detailed information on the reactor configuration and operation conditions can be found in our previous study^7^. From day 193, two regular triangle baffles were installed to prevent short circuits and granular sludge stratification. The first baffle (baffle 1) had a horizontal depth of 2 cm and was installed 13 cm above the base of the reactor. The second baffle (baffle 2), having a height of 1 cm, was installed 29 cm above the base of the reactor (Fig. S8). Liquid and solid samples were collected regularly from different sludge layers along the depth of the SRUSB reactor. Between 9 and 15 samples were taken from each layer during the steady-state operation period (determined by stable COD and sulfate removal). The main physical characteristics of the granular sludge are summarized in Table S1, as described in Hao *et al*.^31^.

### Chemical, physical and biological analysis

Total/soluble COD was examined according to Hao *et al*.^7^. Interference of sulfide was eliminated by adding excess zinc sulfate (ZnSO_4_) forming precipitation of zinc sulfide (ZnS). Three droplets of 10 M NaOH solution were then added to precipitate residual Zn^2+^ as Zn(OH)_2_, followed by centrifugation at 3,500 rpm for 5 min and filtration with 0.45-μm cellulose membrane filter paper (Millipore). The filtrate was then analyzed for total soluble carbonaceous COD^42^. TSS and VSS of the SRUSB were measured following the Standard Methods (2450D and E)^42^. Sulfate and thiosulfate were analyzed using an ion chromatograph (LC-20A, Shimadzu, Japan) equipped with a conductivity detector and an IC-SA2 analytical column. Dissolved sulfide was determined using the methylene blue method^42^, while pH was measured using a multimeter (Multi 3420 with Sentix 940-3 probe, WTW, Germany). Volatile fatty acids (acetate, propionate, butyrate and valeric acids) were analyzed using a 6820 GC System gas chromatograph equipped with a 30 m × 250 μm × 0.25 μm FFAP capillary column (Agilent, USA) and a flame ionization detector. The granular sludge surface hydrophobicity was assessed by adsorption to the organic solvent p-xylene (Merck) according to Rosenberg and Gutnick^43^.

Sludge samples from the middle of the substratum (between the reactor bottom and baffle 1) and superstratum (above the baffle 1) were collected on day 392 to reveal the diversity of the microbial community in the granules, using 454-pyrosequencing of the 16S rRNA gene according to Lee *et al*.^44^ and Zhang *et al*.^45^. Details of the methods for DNA extraction, PCR amplification, pyrosequencing and data analysis are provided in the SI.

### Computational fluid dynamics (CFD) model

The commercial CFD code Fluent 14.0 (ANSYS, USA) based on the finite volume method was employed for the 3-D hydrodynamic simulation of the reactor, with the solid (granular sludge) and liquid (wastewater) phases representing fully interpenetrating continua according to the extended granular flow theory^46^. Turbulence is a three-dimensional phenomenon that cannot be accurately captured with two-dimensional simulations^32^ due to the lack of an appropriate turbulence model and correct empirical constants and closures. Moreover, the Reynolds number of the influent is quite low (Re = 9.6), and therefore a turbulence-free model is commonly assumed and adopted in granular sludge reactor studies^46^,^47^,^48^, which is applied in this study as well.

The interpenetration of the liquid and solid phases in the reactor is dictated by the conservation of mass and momentum. In the solid phase, spherical particles with a narrow size distribution, uniform density and incompressible granules are assumed and modeling was conducted based on the kinetic theory of granular flow. The relevant equations are shown in Table S2 in the SI.

The momentum equation differs for the solid and liquid phases because of pressure variation, in which lift and virtual mass forces are assumed to be negligible. Mass transfer between these two phases is also ignored, such that the transfer forces can be described by empirical drag laws^49^,^50^. The granular sludge bulk viscosity is determined by the equation proposed by Lun *et al*.^51^. The expression of Schaeffer^52^ is further applied for determining the frictional viscosity, with an angle of internal friction of 30°. This angle of internal friction should not affect the results by much^53^. The kinetic part of granular viscosity and conductivity are determined from the relationships given by Syamlal *et al*.^54^. The radial distribution function of Ding and Gidaspow^55^ takes into account the probability of particles colliding with each other when the solid phase becomes densely packed. The detailed initial setting of the CFD model and its key parameters are summarized in Table S3 in the SI.

## Additional Information

**How to cite this article**: Hao, T.-w. *et al*. Example study for granular bioreactor stratification: Three-dimensional evaluation of a sulfate-reducing granular bioreactor. *Sci. Rep*. **6**, 31718; doi: 10.1038/srep31718 (2016).

## Supplementary Material

Supplementary Information

## Figures and Tables

**Table 1:** Abundance of SRB-related genera in the substratum and superstratum.

	*Desulfovibrio*	*Desulfomicrobium*	*Desulfofustis*	*Desulfosarcina*	*Desulfobacter*	*Desulfoulbus*
Substratum	0.22%	0.51%	0.39%	1.02%	7.8%	31.5%
Superstratum	0%	0%	0%	0.45%	1.7%	42.3%

**Table 2:** Performance of different reactor compartments and summary of microbial community shifts.

	0–13 cm	13–30 cm	30–40 cm
pH	5.6 → 7.0	7.0 → 7.7	7.7 → 7.8
Alkalinity (mg CaCO_3_/L)	214 → 560	560 → 573	573 → 548
COD utilization rate	7.3 mg/L/min	10.7 mg/L/min	4.5 mg/L/min
Acidogens Genus abundance	17.6% (3 genera)	13.4% (2 genera)	
SRB genus abundance	41.4% (6 genera)	44.5% (3 genera)	

## References

[b1] Lettinga G., van Velsen A. F. M., Hobma S. W., De Zeeuw W. & Klapwijk A. Use of the upflow sludge blanket (USB) reactor concept for biological wastewater treatment, especially for anaerobic treatment. Biotechnol. Bioeng. 22, 699–734 (1980).

[b2] Liu Y., Xu H. L., Show K. Y. & Tay J. H. Anaerobic technology for wastewater treatment. World J. Microb. Biot. 18, 99–113 (2002).

[b3] Cho S. K. . Low strength ultrasonication positively affects the methanogenic granules toward higher AD performance. Part I: physico-chemical characteristics. Bioresour. Technol. 136, 66–72 (2013). 23563439 10.1016/j.biortech.2013.02.111

[b4] Chen F. Y., Liu Y. Q., Tay J. H. & Ning P. Rapid formation of nitrifying granules treating high-strength ammonium wastewater in a sequencing batch reactor. Appl. Microbiol. Biotechnol. 99, 4445–4452 (2015). 25573473 10.1007/s00253-014-6363-6

[b5] Zheng X. . The long-term effect of nitrite on the granule-based enhanced biological phosphorus removal system and the reversibility. Bioresour. Technol. 132, 333–341 (2013). 23428779 10.1016/j.biortech.2013.01.042

[b6] Speth D. R., in’t Zandt M. H., Guerrero-Cruz S., Dutilh B. E. & Jetten M. Genome-based microbial ecology of anammox granules in a full-scale wastewater treatment system. Nat. Commun. 7, 1–10 (2016). 10.1038/ncomms11172 PMC4821891 27029554

[b7] Hao T., Lu H., Chui H. K., van Loosdrecht M. C. M. & Chen G. H. Granulation of anaerobic sludge in the sulfate-reducing up-flow sludge bed (SRUSB) of SANI^®^ process. Water Sci. Technol. 68, 560–566 (2013a). 23925183 10.2166/wst.2013.182

[b8] Lashkarizadeh M., Munz G. & Oleszkiewicz J. A. Impacts of variable pH on stability and nutrient removal efficiency of aerobic granular sludge. Water Sci. Technol. 73(1), 60–68 (2016). 26744935 10.2166/wst.2015.460

[b9] Ab Halim M. H. . Aerobic sludge granulation at high temperatures for domestic wastewater treatment. Bioresour. Technol. 185, 445–449 (2015). 25851807 10.1016/j.biortech.2015.03.024

[b10] Gonzalez-Estrella J., Sierra-Alvarez R. & Field J. A. Toxicity assessment of inorganic nanoparticles to acetoclastic and hydrogenotrophic methanogenic activity in anaerobic granular sludge. J. Hazard. Mater. 260, 278–85 (2013). 23770618 10.1016/j.jhazmat.2013.05.029

[b11] Zhao Y., Huang J., Zhao H. & Yang H. Microbial community and N removal of aerobic granular sludge at high COD and N loading rates. Bioresour. Technol. 143, 439–446 (2013). 23827440 10.1016/j.biortech.2013.06.020

[b12] Di Felice R., Nicolella C. & Rovatti M. Mixing and segregation in water fluidised-bed bioreactors. Water Res. 31, 2392–2396 (1997).

[b13] Sliekers A. O., Third K. A., Abma W., Kuenen J. G. & Jetten M. S. M. CANON and Anammox in a gas-lift reactor. FEMS Microbiol. Lett. 218, 339–344 (2003). 12586414 10.1016/S0378-1097(02)01177-1

[b14] Winkler M. K. H. . Selective sludge removal in a segregated aerobic granular biomass system as a strategy to control PAO-GAO competition at high temperatures. Water Res. 45, 3291–3299 (2011a). 21513967 10.1016/j.watres.2011.03.024

[b15] Winkler M. K. H., Kleerebezem R., Kuenen J. G., Yang J. & Van Loosdrecht M. C. M. Segregation of biomass in cyclic anaerobic/aerobic granular sludge allows the enrichment of anaerobic ammonium oxidizing bacteria at low temperatures. Environ. Sci. Technol. 45, 7330–7337 (2011b). 21744798 10.1021/es201388t

[b16] Ro K. S. & Neethling J. B. Biological fluidized-Beds containing widely different bioparticles. J. Environ. Eng. Asce. 120, 1416–1426 (1994).

[b17] Safferman S. I. & Bishop P. L. Aerobic fluidized bed reactor with internal media cleaning. J. Environ. Eng.-Asce. 122, 284–291 (1996).

[b18] Gjaltema A., Vinke J. L., van Loosdrecht M. C. M. & Heijnen J. J. Abrasion of suspended biofilm pellets in airlift reactors: Importance of shape, structure, and particle concentrations. Biotechnol. Bioeng. 53, 88–99 (1997). 18629963 10.1002/(SICI)1097-0290(19970105)53:1<88::AID-BIT12>3.0.CO;2-5

[b19] Schreyer H. B. & Coughlin R. W. Effects of stratification in a fluidized bed bioreactor during treatment of metalworking wastewater. Bioeng. Biotechnol. 63, 129–140 (1999). 10.1002/(sici)1097-0290(19990420)63:2<129::aid-bit1>3.0.co;2-o 10099589

[b20] Pena M. R., Mara D. D. & Avella G. P. Dispersion and treatment performance analysis of an UASB reactor under different hydraulic loading rates. Water Res. 40, 445–452 (2006). 16405944 10.1016/j.watres.2005.11.021

[b21] Bayoumi. S. Hydraulic modeling of the liquid flow pattern in a bench-scale UASB reactor. International Water Technology Conference, IWTC11, Sharm El-Sheikh, Egypt. *International Water Technology Association* (*IWTA*). (2007, March 15–18).

[b22] Jiang J. K., Wu J., Zhang J. B., Poncin S. & Li H. Z. Multiscale hydrodynamic investigation to intensify the biogas production in upflow anaerobic reactors. Bioresour. Technol. 155, 1–7 (2014). 24398185 10.1016/j.biortech.2013.12.079

[b23] Wang J. . Microbial community of sulfate-reducing up-flow sludge bed in the SANI process for saline sewage treatment. Appl. Microbiol. Biot. 90, 2015–2025 (2011). 10.1007/s00253-011-3217-3 21494868

[b24] Amaral Filho J., Azevedo A., Etchepare R. & Rubio J. Removal of sulfate ions by dissolved air flotation (DAF) following precipitation and flocculation. Int. J. Miner. Process. 149, 1–8 (2016).

[b25] Van Loosdrecht M. C. M., Brdjanovic D., Chui S. & Chen G. A source for toilet flushing and for cooling, sewage treatment benefits, and phosphorus recovery: direct use of seawater in an age of rapid urbanization. Water 21, 17–19 (2012).

[b26] Hao T. W. . Characterization of sulfate-reducing granular sludge in the SANI^®^ process. Water Res. 47, 7042–7052 (2013b). 24200003 10.1016/j.watres.2013.07.052

[b27] Teaters L. A Computational Study of the Hydrodynamics of Gas-Solid Fluidized Beds. Thesis for the degree of Master of Science in Mechanical Engineering. *Virginia Polytechnic Institute, USA* (2012).

[b28] Neilson A. H. & Allard A. S. Organic Chemicals in the Environment: Mechanisms of Degradation and Transformation 622–623 (Taylor & Francis, 2013).

[b29] Hao T. W. . A review of biological sulfate conversions in wastewater treatment. Water Res. 65, 1–21 (2014). 25086411 10.1016/j.watres.2014.06.043

[b30] Lovely D. R., Dwyer D. F. & Klug M. J. Kinetic analysis of competition of sulfate reducers and methanogens for hydrogen in sediments. Appl. Environ. Microbial. 43, 1373–1379 (1982). 10.1128/aem.43.6.1373-1379.1982 PMC244242 16346033

[b31] Hao T. . Physicochemical and biological characterization of long-term operated sulfate reducing granular sludge in the SANI^®^ process. Water Res. 71, 74–84 (2015). 25600299 10.1016/j.watres.2014.12.051

[b32] Finster K. Microbiological disproportionation of inor-ganic sulfur compounds. J. Sulfur Chem. 29, 281–292 (2008).

[b33] Lu H. . SANI^®^ Process realizes sustainable saline Sewage treatment: steady state model-based evaluation of the pilot scale trial of the process. Water Res. 46, 475–490 (2011). 22130002 10.1016/j.watres.2011.11.031

[b34] Tay J. H., Yang S. F. & Liu Y. Hydraulic selection pressure-induced nitrifying granulation in sequencing batch reactors. Appl. Microbiol. Biotechnol. 59, 332–337 (2002). 12111167 10.1007/s00253-002-0996-6

[b35] Ren T. T., Mu Y., Liu L., Li X. Y. & Yu H. Q. Quantification of the shear stresses in a microbial granular sludge reactor. Water Res. 43, 4643–4651 (2009). 19647851 10.1016/j.watres.2009.07.019

[b36] Diamantis V. I. & Aivasidis A. Comparison of single- and two-stage UASB reactors used for anaerobic treatment of synthetic fruit wastewater. Enzyme Microb. Technol. 42, 6–10 (2007).

[b37] Aivasidis A. & Diamantis V. Biochemical reaction engineering and process development in anaerobic wastewater treatment. Adv. Biochem. Eng./Biotechnol. 92, 49–76 (2005). 10.1007/b98919 15791932

[b38] van Houten R. T., van der Spoel H., van Aelst A. C., Hulshoff Pol L. W. & Lettinga G. Biological sulfate reduction using synthesis gas as energy and carbon source. Biotechnol Bioeng. 20, 136–144 (1996). 18626930 10.1002/(SICI)1097-0290(19960420)50:2<136::AID-BIT3>3.0.CO;2-N

[b39] Cock S. L. & de Stouvenel R. A. Lactic acid production by a strain of Lactococcus lactis subs lactis isolated from sugar cane plants. Electron J. Biotechn. 9, 40–45 (2006).

[b40] Villa Gómez D. K. Simultaneous sulfate reduction and metal precipitation in an inverse fluidized bed reactor. *PhD thesis, UNESCO-IHE Institute for Water Education, Delft, the Netherlands* (2013).

[b41] Anil K. P., Srinivas T. N. R., Sasikala C., Venkata R. C. & Suling J. I. J. Prosthecochloris indica sp. Nov., a novel green sulfur bacterium from a marine aquaculture pond, Kakinada, India. J. Gen. Appl. Microbiol. 55, 163–169 (2009). 19436133 10.2323/jgam.55.163

[b42] APHA. Standard Methods for the Examination of Water and Wastewater. 21st edn, American Public Health Association (2005).

[b43] Rosenberg M., Gutnick D. & Rosenberg E. Adherence of bacteria to hydrocarbons: A simple method for measuring cell-surface hydrophobicity. FEMS Microbiol Lett. 9, 29–33 (1980).

[b44] Lee O. O. . Pyrosequencing reveals highly diverse and species-specific microbial communities in sponges from the Red Sea. The ISME journal 5, 650–664 (2011). 21085196 10.1038/ismej.2010.165 PMC3105750

[b45] Zhang T., Shao M. F. & Ye L. 454 Pyrosequencing reveals bacterial diversity of activated sludge from 14 sewage treatment plants. The ISME journal 6, 1137–1147 (2012). 22170428 10.1038/ismej.2011.188 PMC3358032

[b46] Cornelissen J. T., Taghipour F., Escudié R., Ellis N. & Grace J. R. CFD modelling of a liquid–solid fluidized bed. Chem. Eng. Sci. 62, 6334–6348 (2007).

[b47] Fan L., Grace J. R. & Epstein N. CFD simulation of a liquid-fluidized bed of binary particles. The 13th International Conference on Fluidization-New Paradigm in Fluidization Engineering. Gyeong-ju, Korea. *ECI Digital Archives*. (2010, May 16–21).

[b48] Gidaspow D. & Lu H. A comparison of gas–solid and liquid–solid fluidization using kinetic theory and statistical mechanics. (Eds Fan L. S., Knowlton T. M.) 661–668 (Engineering Foundation, 1998).

[b49] Wen C. Y. & Yu Y. H. Mechanics of fluidization. Chemical Engineering Progress Symposium Series 62, 100–111 (1966).

[b50] Gidaspow D., Bezburuah R. & Ding J. Hydrodynamics of circulating fluidized beds, kinetic theory approach. (Eds Potter O. E., Nicklin D. J.) 75–82 (Engineering Foundation, 1992).

[b51] Lun C. K. K., Savage S. B., Jeffrey D. J. & Chepurniy N. Kinetic theories for granular flow: inelastic particle in Couette flow and slightly inelastic particles in a general flow field. J. Fluid. Mech. 140, 223–256 (1984).

[b52] Schaeffer D. G. Instability in the evolution equations describing incompressible granular flow. J. Differ. Equations 66, 19–50 (1987).

[b53] van Wachem B. G. M., Schouten J. C., van den Bleek C. M., Krishna R. & Sinclair L. L. Comparative analysis of CFD models of dense gas–solid systems. A. I. Ch. E. Journal 47, 1035–1051 (2001).

[b54] Syamlal M., Rogers W. & O’Brien T. J. MFIX Documentation: Theory Guide. Technical Note. Available at: https://mfix.netl.doe.gov/documentation/Theory.pdf (Accessed: 17th October 2014) (1993).

[b55] Ding J. & Gidaspow D. A. A bubbling fluidization model using kinetic theory of granular flow. A.I.Ch.E. Journal 36, 523–538 (1990).

