# Supplementary material for: Example study for granular bioreactor stratification: Three-dimensional evaluation of a sulfate-reducing granular bioreactor
Source: Sci Rep. 2016 Aug 19;6:31718. doi: 10.1038/srep31718 (PMC4990961; doi:10.1038/srep31718)
Supplement: Supplementary Information [file srep31718-s1.pdf]

Supplementary Information for

Example study for granular bioreactor stratification: three dimensional evaluation of a sulfate reducing granular bioreactor

Tian-wei Hao[1], Jing-hai Luo[1], Kui-zu Su[2], Li Wei[1*], Hamish R. Mackey[3], Kun Chi[1], and Guang-Hao Chen[1,4,5*]

[1] Department of Civil & Environmental Engineering, The Hong Kong University of Science and Technology, Clear Water Bay, Kowloon, Hong Kong.

[2]School of Civil Engineering and Water Conservancy, Hefei University of Technology, Hefei, China

[3]College of Science and Engineering, Hamad bin Khalifa University, Education City, Doha, Qatar

[4]Water Technology Lab, The Hong Kong University of Science and Technology, Clear Water Bay, Kowloon, Hong Kong, China.

[5]Hong Kong Branch of Chinese National Engineering Research Center for Control & Treatment of Heavy Metal Pollution, HKUST, Clear Water Bay, China

*corresponding author: ceghchen@ust.hk

Number of pages: 10

Number of tables: 4

Number of figures: 8

Methods of biological analysis

Barcoded universal bacteria primers

Forward primer 515F and reverse primer 926R

(F515:5'-CCATCTCATCCCTGCGTGTCTCCGACTCAGCAGAGTCTGTGCCAG CMGCCGCGGTAA -3'

926R:5'-CCTATCCCCTGTGTGCCTTGGCAGTCTCAGCCGTCAATTYYTTTRA GTTT -3'),

Polymerase chain reaction (PCR) amplification and pyrosequencing

Fragments of the 16S rDNA gene were amplified by PCR using barcoded universal bacteria primers 515F and 926R targeting the V4 and V5 hypervariable regions (Quince et al., 2011). The average length of PCR product is 423 bp.

The 100-μl PCR reaction mixture contained 5 U of P*fu* Turbo DNA polymerase (Stratagene, La Jolla, CA, USA), 1X P*fu* reaction buffer, 0.2 mM of dNTPs (TaKaRa, Dalin, China), 0.1 μM of each barcoded primer, and 20 ng of genomic DNA template. PCR was performed under the following thermocycle: 94 ºC for 5 min followed by 30 cycles of 94 ºC for 30 s, 53 ºC for 30 s and 72 ºC for 45 s, and a final extension at 72 ºC for 10 min.

Pyrosequencing reads with ambiguous nucleotides, shorter than 200-nucleotides or without a complete barcode and primer at one end were removed and excluded from further analysis. The quality filtered reads were denoised by flowgram clustering to remove the homopolymer errors (Reeder and Knight, 2010).

Each sample was sequenced three times and the results with high quantity and

relatively even fragments were chosen for further analysis. Total of eight samples were pooled for pyrosequencing analysis at each time.

Sequence analysis

Raw sequence data was firstly processed by trimming barcode tags and primer sequences. FASTA files were generated from the resultant sequences according to the barcodes of individual samples. The sequences were then aligned using the software Mothur ver. 1.17.0 (Schloss et al., 2009) and the distance matrix was produced. Operational taxonomic unit (OTU) was determined at the 90, 95 and 97% similarity levels (Mothur v. 1.17.0). Rarefaction curves were determined based on the calculated OTUs. For the taxonomy-based analysis, the representative sequences from each OTU were subjected to the RDP-II Classifier of the Ribosomal Database Project (RDP) (Cole et al., 2009).The relative abundance and occurrence of tags assigned to different taxonomies were visualized as a heatmap using the software MeV 4.8.1.

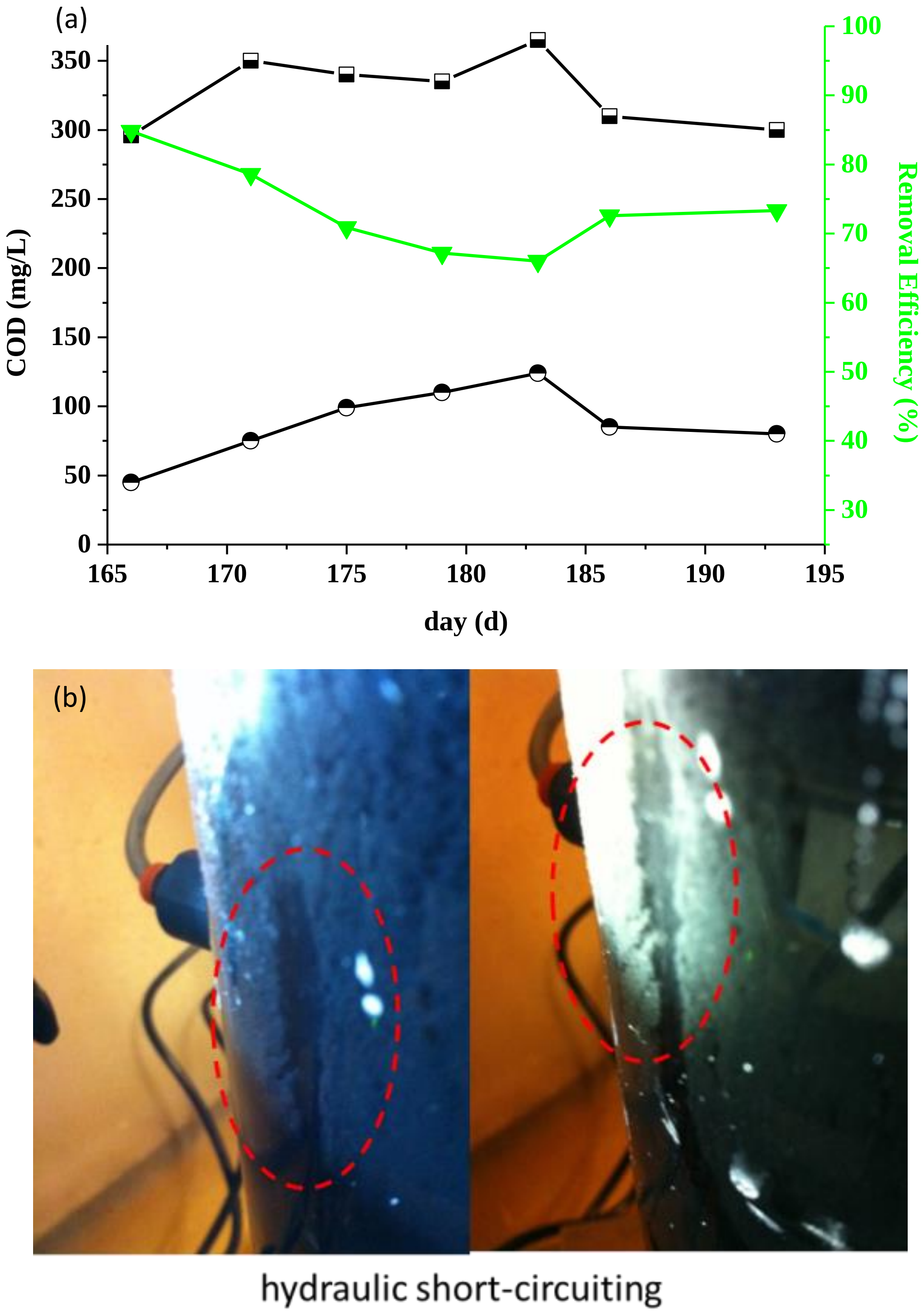

Figure S1. (a) Deterioration of COD removal efficiency in UASB from day 160 and (b) observed short-circuiting in the reactor

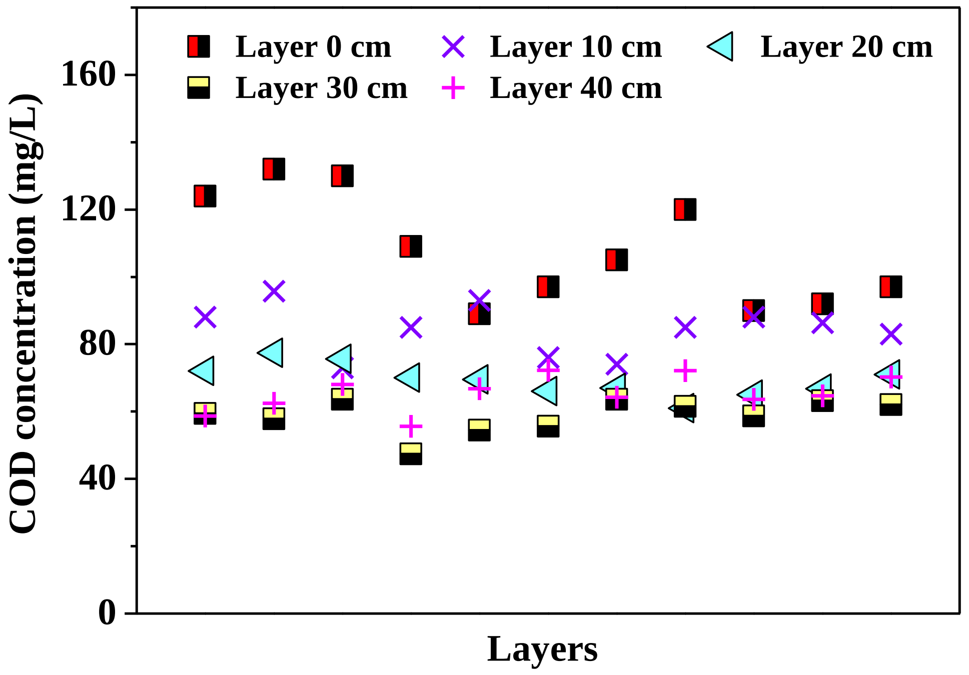

Figure S2. Soluble COD concentration profiles at different sludge layers

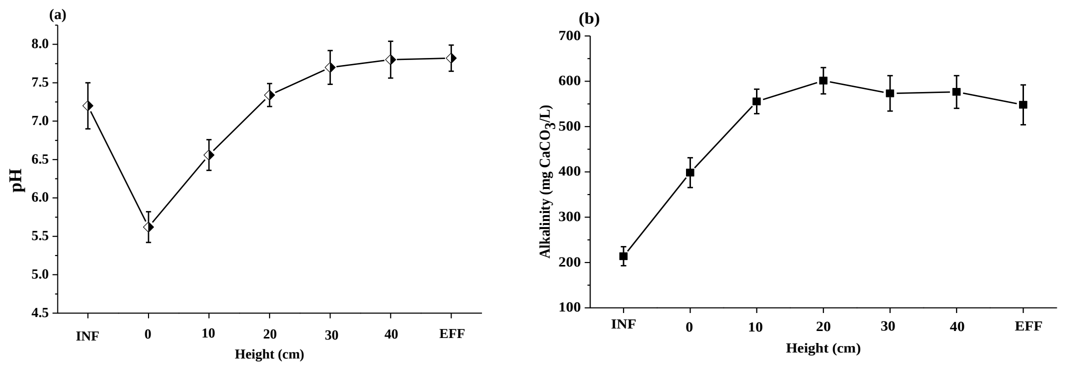

Figure S3. The pH (a) and alkalinity (b) variation profile along the height of the SRUSB

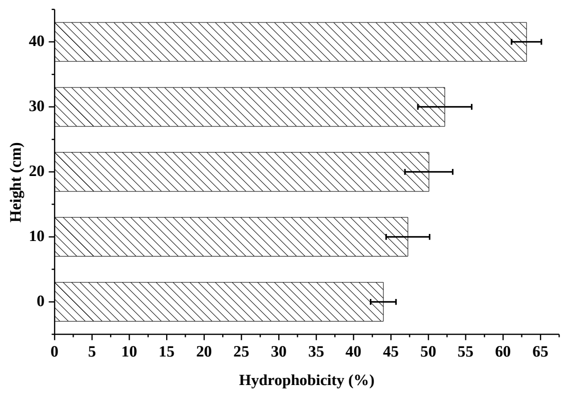

Figure S4. Hydrophobicity profile of sludge surface at different sludge layers

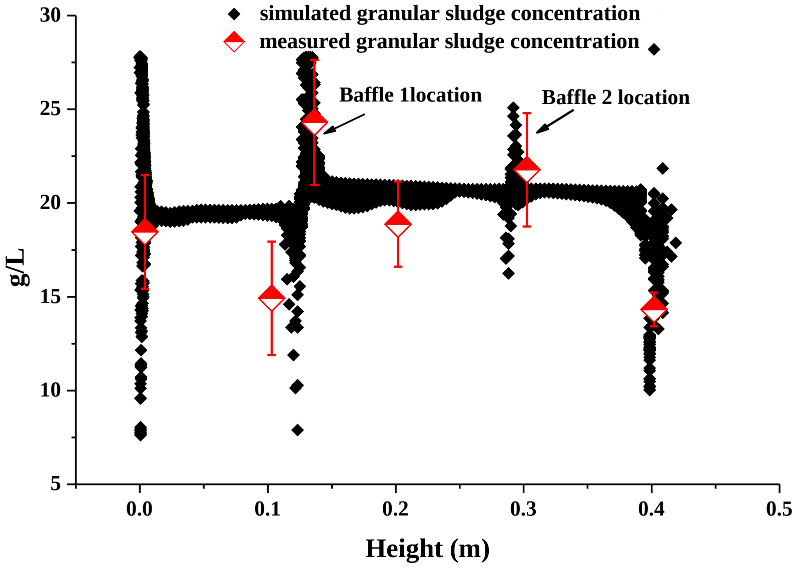

Figure S5. The simulated and measured concentration profile of the granular sludge along the SRUSB height (g TSS/L).

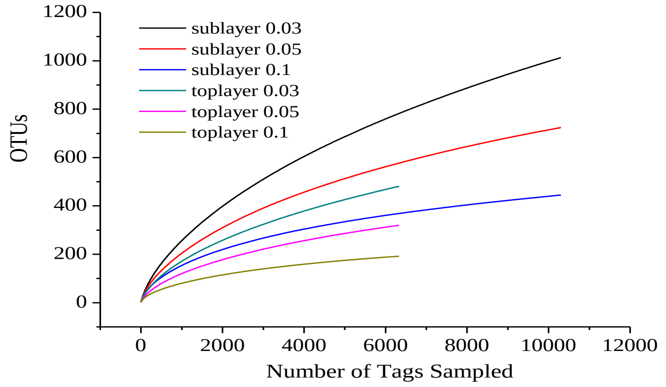

Figure S6. Rarefaction curves of 454 pyrosequencing

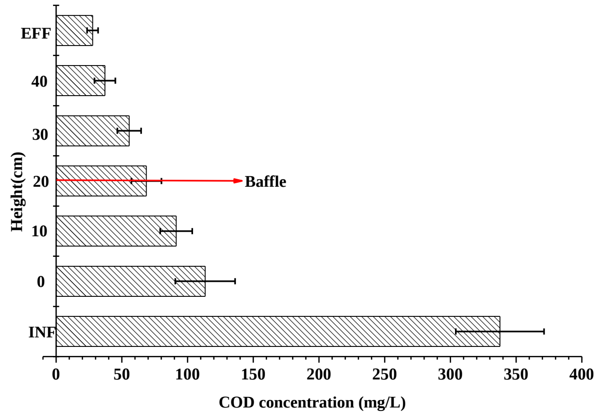

Figure S7. COD degradation profile along the SRUSB axis after baffle optimization

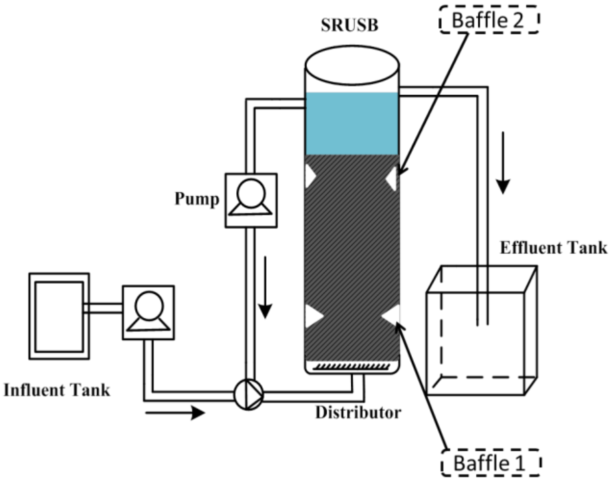

Figure S8. Schematic of SRUSB after two baffles installation at day 193

Table S1. Main physical characteristics of the SRB granules in the steady-state SRUSB.

VSS/TSS	Diameter (μm)	SVI_5 (ml/g)	Specific gravity	Settling velocity (m/h)
72%	420−450	~30	1.067−1.074	18−65

VSS: volatile suspended solid; TSS: total suspended solid

Table S2. Relevant model equations in Fluent 14.0 CFD code

Continuity equation	$\frac{\partial}{\partial_t}(\alpha_q\rho_q) + \nabla\cdot(\alpha_q\rho_q\,\vec{v_q}) = 0$	
Momentum equations	$\frac{\partial}{\partial_t}(\alpha_l\rho_l\,\vec{v_l}) + \nabla\cdot(\alpha_q\rho_q\,\vec{v_q}^{\,2}) = -\alpha_l\nabla p + \nabla\cdot\bar{\bar{\tau}}_l + \alpha_l\rho_l\,\vec{g} + K_{sl}(\vec{v_s} - \vec{v_l})$	
	$\frac{\partial}{\partial_t}(\alpha_s\rho_s\,\vec{v_s}) + \nabla\cdot(\alpha_s\rho_s\,\vec{v_s}^{\,2}) = -\alpha_s\nabla p + \nabla\cdot p_s + \alpha_s\rho_s\,\vec{g} + K_{ls}(\vec{v_l} - \vec{v_s})$	
	$\bar{\bar{\tau}}_q = \alpha_q\mu_q(\nabla\cdot\vec{v_q} + \nabla\cdot\vec{v_q}^{\,T}) + \alpha_q(\lambda_q - \frac{2}{3}\mu_q)\nabla\cdot\vec{v_q}\bar{\bar{I}}$	
Liquid solid drag	$K_{sl} = \frac{3}{4}C_D\frac{\alpha_s\alpha_l\rho_l\lvert\vec{v_s} - \vec{v_l}\rvert}{d_s}\alpha_l^{\;-2.65} \quad \alpha_l$	Wen and Yu (1966)
	$K_{sl} = \begin{cases} \frac{3}{4}C_D\frac{\alpha_s\alpha_l\rho_l\lvert\vec{v_s} - \vec{v_l}\rvert}{d_s}\alpha_l^{\;-2.65} & \alpha_l > 0.8 \\ 150\frac{\alpha_s(1-\alpha_l)\mu_l}{\alpha_l {d_s}^2} + 1.75\frac{\alpha_s\rho_l\lvert\vec{v_s} - \vec{v_l}\rvert}{d_s} & \alpha_l \le 0.8 \end{cases}$	Gidaspow et al. (1992)
	$C_D = \frac{24}{\alpha_l Re_s}[1 + 0.15(\alpha_l Re_s)^{0.687}]$	
	$Re_s = \frac{d_s\rho_l\lvert\vec{v_s} - \vec{v_l}\rvert}{\mu_l}$	
Solids pressure	$p_s = \alpha_s\rho_s\theta_s + 2\rho_s(1 + e_{ss})\alpha_s g_{0,ss}\theta_s$	Lun et al. (1984)
Radial distribution function	$g_0 = [1 - (\frac{\alpha_s}{\alpha_{s,max}})^{\frac{1}{3}}]^{-1}$	Ding and Gidaspow (1990)
Solids shear stress	$\mu_s = \mu_{s,col} + \mu_{s,kin} + \mu_{s,fr}$	
	$\mu_{s,col} = \frac{4}{5}\alpha_s\rho_s d_s g_{0,ss}(1 + e_{ss})\sqrt{\frac{\Theta_s}{\pi}}$	Gidaspow et al. (1992)
	$\mu_{s,kin} = \frac{\alpha_s d_s\rho_s\sqrt{\pi\Theta_s}}{6(3 + e_{ss})}\left[1 + \frac{2}{5}(1 + e_{ss})(3e_{ss} - 1)\alpha_s g_{0,ss}\right]$	Syamlal et al. (1993)
	$\mu_{s,fr} = \frac{\rho_s\sin\Phi}{2\sqrt{I_{2D}}}$	Schaeffer (1987)
Bulk viscosity	$\lambda_s = \frac{4}{5}\alpha_s d_s\rho_s g_{0,ss}(1 + e_{ss})\sqrt{\frac{\Theta_s}{\pi}}$	Lun et al. (1984)
Granular temperature	$\frac{3}{2}\left[\frac{\partial}{\partial t}(\rho_s\alpha_s\Theta_s) + \nabla.(\rho_s\alpha_s\bar{v}_s\Theta_s)\right] =$	
	$(-\rho_s\bar{\bar{I}} + \bar{\bar{\tau}}_s):\nabla.\bar{v}_s + \nabla.(k_{\theta s}\nabla\Theta_s) - \gamma_{\theta s} + \Phi_{ls}$	
	$k_{\theta s} = \frac{15 d_s\rho_s\alpha_s\sqrt{\Theta_s\pi}}{4(41 - 33\eta)}\left[1 + \frac{12}{5}\eta^2(4\eta - 3)\alpha_s g_{0,ss} + \frac{16}{15\pi}(41 - 3\eta)\eta\alpha_s g_{0,ss}\right]$	Syamlal et al. (1993)
	$\eta = \frac{1}{2}(1 + e_{ss})$	
	$\gamma_{\theta s} = \frac{12(1 - e_{ss}^2)g_{0,ss}}{d_s\sqrt{\pi}}\rho_s\alpha_s^2\Theta_s^{3/2}$	Lun et al. (1984)
	$\Phi_{ls} = -3k_{ls}\Theta_s$	
Packing limit	$\alpha_{s,max} = \frac{\rho_b}{\rho_s}$	

Table S2-1. Definition of symbol in table A1.

Symbol	Description	Units
Alphabetic		
C_D	drag coefficient.	dimensionless
d	diameter,	m
e	coefficient of restitution,	dimensionless
g	gravitational acceleration,	m/s^2
g0	radial distribution coefficient,	dimensionless
$k_{\theta s}$	diffusion coefficient for granular energy,	dimensionless
K	interphase exchange coefficient,	dimensionless
P	pressure,	Pa
Re	Reynolds number,	dimensionless
t	time,	s
Greek letters		
α	volume fraction,	dimensionless
$\gamma_{\theta s}$	collision dissipation of energy,	kg/s^3m
η	dynamic viscosity,	Pas
θ	granular temperature,	m^2/s^2
$\bar{\bar{I}}$	stress tensor,	dimensionless
I_{2D}	second invariant of the deviatoric stress tensor,	dimensionless
λ	bulk viscosity,	Pas
μ	shear viscosity,	Pas
ν	velocity,	m/s
ρ	density,	kg/m^3
$\bar{\bar{\tau}}$	stress tensor,	Pa
φ	angle of internal friction,	deg
Φ	Transfer rate of kinetic energy,	kg/s^3m
Subscripts		
col	collision,	dimensionless
fr	friction,	dimensionless
kin	kinetic,	dimensionless
l	liquid phase,	dimensionless
max	maximum value,	dimensionless
q	either liquid or solid phase,	dimensionless
s	solid phase,	dimensionless

Table S3. Base case simulation settings

Description	Base case setting/value
Mesh size, time step, convergence criteria and discretization method	
Mesh resolution	602464
Convergence criteria	10^{-3}
Maximum iterations	30
Discretization method	First order upwind
Time step	0.01
Geometry, boundary, initial and operating conditions	
Bed width	88 mm
Bed length	450 mm
Initial bed height	310 mm
Initial solids packing	0.60
Outlet boundary condition	Pressure outlet
Wall boundary condition	No slip (liquid) condition
Gravitational acceleration	9.81 m/S^2
Operating pressure	1.013x10^5 pa
Liquid superficial velocity	7.4774e-4 m/s
Inlet boundary condition	Uniform velocity inlet
Liquid properties	
At room temperature	298 K
viscosity	0.001003 Pa s
density	998.2 kg/m^3
Granular sludge properties	
Mean diameter	0.42 mm
density	1130 kg/m^3
Packed bed solids volume fraction	0.60
Initial inventory	1.2279 kg

Reference

Cole, J. R., Wang, Q., Cardenas, E., Fish, J., Chai, B., Farris, R. J., Kulam-Syed-Mohideen, A. S., McGarrell, D. M., Marsh, T., Garrity, G. M. & Tiedje, J. M. The Ribosomal Database Project: improved alignments and new tools for rRNA analysis. *Nucleic Acids Research.* **37,** 141–145 (2009).

Ding, J. & Gidaspow, D. A. A bubbling fluidization model using kinetic theory of granular flow. *A.I.Ch.E. Journal.* **36,** 523–538 (1990).

Gidaspow, D., Bezburuah, R. & Ding, J. Hydrodynamics of circulating fluidized beds, kinetic theory approach. *In: Potter, O.E., Nicklin, D.J. (Eds.), Fluidization VII. Engineering Foundation, New York.* 75–82 (1992).

Lun, C. K. K., Savage, S. B., Jeffrey, D. J. & Chepurniy, N. Kinetic theories for granular flow: inelastic particle in Couette flow and slightly inelastic particles in a general flow field. *Journal of Fluid Mechanics*. **140,** 223–256 (1984).

Quince, C., Lanzen, A., Davenport, R. & Turnbaugh, P. J. Removing noise from pyrosequenced amplicons. *BMC Bioinformatics.* **12,** 38–56 (2011).

Reeder, J. & Knight, R. Rapidly denoising pyrosequencing amplicon reads by exploiting rank-abundance distributions. *Nature Methods.* **7,** 668–669 (2010).

Schloss, P. D., Westcott, S, L,, Ryabin, T., Hall, J. R., Hartmann, M., Hollister, E. B., Lesniewski, R. A., Oakley, B. B., Parks, D. H., Robinson, C. J., Sahl, J. W., Stres, B., Thallinger, G. G., Van Horn, D. J. & Weber, C. F. Introducing mothur: open-source, platform-independent, community-supported software for describing and comparing microbial communities. *Applied and Environmental Microbiology*. **75,** 7537–41 (2009).

Schaeffer, D. G. Instability in the evolution equations describing incompressible granular flow. *Journal of Differential Equations.* **66,** 19–50 (1987).

Syamlal, M., Rogers, W. & O'Brien, T. J. MFIX Documentation: Theory Guide. National Technical Information Service, vol. 1. *Springfield, VA, DOE/METC-9411004, NTIS/DE9400087.* (1993).

Wen, C. Y. & Yu, Y. H. Mechanics of fluidization. *Chemical Engineering Progress Symposium Series.* **62,** 100–111(1966).
